# Stoichiometric estimates of the biochemical conversion efficiencies in tsetse metabolism

**DOI:** 10.1186/1472-6785-5-6

**Published:** 2005-08-05

**Authors:** Adrian V Custer

**Affiliations:** 1 Department of Environmental Science, Policy and Management, 201 Wellman Hall #3112, University of California, Berkeley, Berkeley, CA 94720, USA

## Abstract

**Background:**

The time varying flows of biomass and energy in tsetse (*Glossina*) can be examined through the construction of a dynamic mass-energy budget specific to these flies but such a budget depends on efficiencies of metabolic conversion which are unknown. These efficiencies of conversion determine the overall yields when food or storage tissue is converted into body tissue or into metabolic energy. A biochemical approach to the estimation of these efficiencies uses stoichiometry and a simplified description of tsetse metabolism to derive estimates of the yields, for a given amount of each substrate, of conversion product, by-products, and exchanged gases. This biochemical approach improves on estimates obtained through calorimetry because the stoichiometric calculations explicitly include the inefficiencies and costs of the reactions of conversion. However, the biochemical approach still overestimates the actual conversion efficiency because the approach ignores all the biological inefficiencies and costs such as the inefficiencies of leaky membranes and the costs of molecular transport, enzyme production, and cell growth.

**Results:**

This paper presents estimates of the net amounts of ATP, fat, or protein obtained by tsetse from a starting milligram of blood, and provides estimates of the net amounts of ATP formed from the catabolism of a milligram of fat along two separate pathways, one used for resting metabolism and one for flight. These estimates are derived from stoichiometric calculations constructed based on a detailed quantification of the composition of food and body tissue and on a description of the major metabolic pathways in tsetse simplified to single reaction sequences between substrates and products. The estimates include the expected amounts of uric acid formed, oxygen required, and carbon dioxide released during each conversion. The calculated estimates of uric acid egestion and of oxygen use compare favorably to published experimental measurements.

**Conclusion:**

This biochemical analysis provides reasonable first estimates of the conversion efficiencies for the major pathways used by tsetse metabolism. These results now enable a deeper analysis of tsetse ecology based on the construction of a dynamic mass-energy budget for tsetse and their populations.

## Background

The dynamics of mass and energy flows in tsetse (*Glossina *Wiedemann), the time-varying rates of biomass ingestion and use, influence most aspects of the ecology of these flies including the nutrition and growth of individuals, the intrinsic growth rate of tsetse populations, and the transmission dynamics of the tsetse vectored trypanosomiases. A particularly effective approach to the examination of these dynamics involves the assembly and evaluation of a dynamic mass-energy budget [1-3] constructed specifically for tsetse. Such a budget could capture the essence of the time varying flows of biomass and energy in tsetse, could explain these dynamics mechanistically based on the internal physiology of the flies, and could form the core of a physiologically based model of tsetse population dynamics [4-6]. The budget and population model could, in turn, form the core of an epidemiological model of trypanosomiasis dynamics [7,8] because the tsetse vectored trypanosomiases are transmitted when tsetse feed to acquire biomass and energy.

At the center of any dynamic mass-energy budget applicable to heterotrophs lies the conversion of food substrates into usable products, either body tissue or metabolic energy. The efficiencies of these conversions influence the rates of mass and energy flow since the rate of food intake must at least equal, but may exceed, the rate of end product use divided by the conversion efficiency. Food conversion involves, first, the breakdown of food substances leading either to their absorption or to their egestion and, then, the post-absorptive processing of nutrients leading either to their assimilation as body mass, to their consumption as energy, or to their excretion either because they are too costly to process or because they could potentially accumulate to toxic levels. The ultimate yield from biomass conversion will reflect the richness of the food source, the loss of food which is not digested, the loss of resources which are either directly egested or absorbed but then excreted because they are too costly to assimilate, the costs involved in conversion, the efficiency of the conversion process, the cost of removal of potentially toxic substances in the food, the costs of transportation and growth necessary to digestion, and the energetic richness of the resulting substances.

This paper develops quantitative estimates of the efficiencies of biomass conversion in tsetse. These conversion estimates will provide the working numbers needed to elucidate tsetse energetics and thereby construct a dynamic mass-energy budget which can quantify the need for food of individual flies based on their rates of respiration, development, growth, and reproduction.

### Approaches to estimation

The fundamental principles of mass and energy conservation constrain the efficiency of conversion in metabolic reactions to lie at or below unity. Other principles from thermodynamics, physics, chemistry, and biochemistry then successively lower the maximum theoretical yield by including the impact of more inefficiencies and costs, thereby achieving more realism and constraining the estimate sequentially closer to the actual biological efficiency experienced by the organism.

The balance of mass can be treated separately from the balance of energy since biological systems do not involve nuclear transformations but the two balances are coupled so they can be leveraged jointly for estimation. The physical law of mass conservation, when applied at the organismal level, requires that, across any time interval, the original mass of the organism plus the mass of food material ingested must equal the final mass of the organism plus the mass of material egested. This was the analytic approach of van Helmont's famous 1648 willow growth experiment [9]. Chemistry refines the law of mass conservation into a law of element balance in which the masses of each element can be treated separately and each must balance across all the biological reactions. This approach is widely used in ecology notably in the field of ecological stoichiometry [10] and in the recent studies of isotope flows. The biochemical approach further refines the estimate by considering the fate of specific structural molecular fragments. This is the approach that is required for nutritional studies of the trophic flow of vitamins or of necessary and non-synthesizable amino acids. The biological approach refines the estimate even further by considering explicitly indigestible tissue structures such as the seeds in fruit. The strategy of mass balance provides a useful component for the estimation of conversion yields because each of these approaches defines a strict equality between the start and the end of the conversion, albeit with successively more detail in the entity being balanced.

The balance of energy in the conversion process can also be used in estimating the efficiency of conversion. While a full energetic budget would consider the radiation balance of organisms as well as the balance in the potential energy of chemical bonds, this study focuses on the balance of chemical energy only, because the biologically useful energy is exclusively that of chemical bonds and because, in heterotrophs, the radiation budget only affects the heat balance of the organism. The omission of radiation from the energy budget alters the analysis from one of a strict equality between the inputs and outputs to one of a constraining inequality because the energy lost as heat is not quantified. The laws of thermodynamics place an upper limit on the efficiency of energetic conversion: the first law requires that the conversion product have at most the same amount of energy as the substrate while the second law states that the efficiency of conversion must be less than unity. Since the potential energy of chemical bonds is only accessible by conversion of the original bonds to new bonds, the approach of physical chemistry examines the potential energy available from the complete oxidation of the food and body tissue with a suitable oxidant. This is the approach taken by the studies which use calorimeters to burn food, exuvium, and body tissues in oxygen and then calculate the metabolic efficiency of energy conversion based on the difference in potential chemical energy. This approach has been used to obtain a static energy budget for tsetse [11]. The approach of biochemistry reduces the estimate of conversion efficiency from that provided by physical chemistry because it considers explicitly the energy recovered through the reactions of biochemical conversion rather than the energy potentially recoverable from the overall chemical reaction. The difference between the two can be significant with the core reactions involved in pyruvate catabolism only capturing, at a maximum, 33% of the chemically available energy [12]. The biological approach further reduces the estimate of energetic recovery efficiency by considering the inefficiencies of conversion due to the imperfection of biological structures and by including the biological costs of digestion, of transport, and of storage. The biological approach would, for example, consider the inefficiencies in the production of ATP due to leakage of the mitochondrial membrane and the costs of maintenance of the digestive lumen, formation of digestive enzymes, manufacture of metabolic enzymes, formation of new cells for the growth of internal organs used in digestion [13-15], and synthesis of molecular complexes for transport such as the joining of glycerides to proteins. However, these biological inefficiencies and costs do not necessarily need to be considered separately. Biological inefficiencies, if they are static or have reasonable average values, can be incorporated directly into the stoichiometric estimation process. Biological costs, to the extent that they involve inhalation of oxygen or exhalation of carbon dioxide, can be accounted for as separate costs in an energetic budget rather than explicitly included in the estimate of conversion efficiency.

Experimental approaches to the estimation of conversion yields have not yet obtained estimates for the major biomass conversions in multicellular animals. Experiments on whole organisms have examined the conversion costs as part of the 'work of digestion', alternatively called the 'specific dynamic action', 'feeding heat increment', or 'postprandial thermogenesis'. These studies use calorimetric measurements and measurements of post-digestive respiration to derive estimates of the costs of digestion in metabolic energy production. A study of this kind was able to estimate that the costs of digestion in a python species account for around a third of the energy available in the food [13]. More detailed experiments are attempting to resolve the different costs but these studies have not yet separated biological costs from the biochemical costs nor, apparently, have such experiments yet distinguished the costs of body tissue formation from the costs of metabolic energy production. Experiments on single cells are achieving remarkable results and now aim to model the full chemical kinetics to estimate the actual production yields of specific metabolic products, especially those which are industrially important. Such experiments are beginning to determine quantitatively the realized conversion efficiencies based on measurements of the flux of the ^13^C isotope and drawing on the use of mass spectrometry, gas chromatography, and nuclear magnetic resonance detection [16,17]. However, these conversion estimates depend extensively on coupled stoichiometric modeling [18,19] so these are not simply experimental approaches but rather integrations of experimental and theoretical strategies.

### This analytic approach

This paper derives quantitative estimates of upper theoretical yields for each of the major conversions in tsetse metabolism using essentially a biochemical approach. The approach was chosen because it could rapidly obtain a complete set of estimates based on a mechanistic interpretation and do so with more accuracy than alternative approaches. The biochemical approach promised to be rapid since it could be performed solely through calculation; indeed, the early success of an initial quick study led to its expansion into this complete analysis. Because the scientific literature now provides all the data required for the analysis, including the composition of tsetse food and tissues, the metabolic pathways, and the reaction sequences, the biochemical approach is able to obtain a complete set of estimates for each of the major conversions. The biochemical approach also provides a mechanistic basis which can explain the yield estimates as the sum of yields and costs in component reactions rather than being simply an empirical measure derived from experiment. The biochemical approach is expected to provide a more accurate estimate than would a calorimetric approach because it considers explicitly more of the costs and inefficiencies of the conversion process.

The calculations presented in this work are necessary because no applicable preexisting results could be found. Tsetse metabolism is unusual in combining a protein diet, the use of uric acid for the disposal of nitrogen, and the use of the amino acid proline as the primary substrate for energy production during flight. These peculiarities bring difficulties to the reuse of existing stoichiometric calculations. The reference values which would be necessary to avoid the calculations presented in this paper could not be found despite consultations with physiologists specializing in bioenergetics and a search of the general literature. The literature on tsetse energetics also fails to provide suitable values. The most complete analysis of tsetse metabolic energetics is the static energy budget presented by Bursell and Taylor [11] which integrates most of the earlier research. That paper presents empirical estimates for both the conversion efficiency of blood to body fat and the consumption of oxygen during fat catabolism but does not provide estimates for the conversion efficiencies of the other major metabolic pathways.

This calculation of conversion yields involves several steps. Tsetse metabolism is simplified down to its core pathways. Tsetse food and body tissues are reduced to simple representative compositions. Each metabolic pathway is expanded into a single series of reactions which lead from the composition of the substrates to that of the products and by-products. Metabolic reactions are considered to occur in a steady state system in which a single input is entirely converted and the system restored to its original state. Based on this view, the yields can be derived from simple stoichiometric calculations. This approach derives estimates of the upper theoretical yields for each of the metabolic conversions considered important in tsetse metabolism and obtains estimates of the uric acid formed to dispose of nitrogenous waste, of the oxygen required, and of the carbon dioxide released in each conversion.

This biochemical approach to the calculation of conversion yields has several conceptual consequences. This approach implicitly considers conversion as a fixed process with static conversion efficiencies since the approach describes, for each conversion, only the single, most efficient pathway, and any such reaction sequence will follow Avogadro's principle of fixed numerical proportions between the substrates and products of chemical reactions. Since tsetse feed on a single, invariant food substrate, namely vertebrate blood [20,21], this analysis does not consider changing efficiencies of metabolism due to food switching or nutritional variation. This greatly simplifies the analysis and budgeting of tsetse energetics by allowing a fixed conversion rate between food and products, for example, an adult fly body mass can be explicitly quantified in terms of its bloodmeal equivalents. Another consequence of the approach is that the conversion pathways are implicitly considered independent of each other.

### A simplified description of tsetse metabolism

This analysis simplifies tsetse metabolism to the conversions which account for the bulk of the transformations of food into metabolic energy, of food into body mass, and of storage body mass into metabolic energy.

Tsetse feed uniquely on vertebrate blood which is composed of approximately 80% water and 20% protein. The amounts of lipids, carbohydrates, and nucleic acids in blood are negligible, between 0.8% and 1% of wet blood or 4% and 5% of the dry weight [22,23], so tsetse can be assumed, as a first approximation, to feed exclusively on protein. Tsetse egest haematin unprocessed but digest the remaining blood protein, absorbing the constituent amino acids into their haemolymph [24,25]. Therefore, the tsetse food source consists essentially of a pool of amino acids which are converted into metabolic energy or into tsetse body tissue.

Metabolic energy is provided by a number of molecules including the reducing molecules nicotinamide-adenine dinucleotide (hereinafter NADH) and flavin-adenine dinucleotide (FADH_2_) and including adenosine triphosphate (ATP). These each have other closely related molecules with essentially identical roles in metabolism. In this paper, all these energetic molecules are treated for simplicity as their equivalent amount of ATP.

Tsetse body tissue composition is variable but is approximately 70% water, 10% ether soluble dry weight, and 20% residual dry weight (RDW) [26,27]. The ether soluble fraction of dry weight is assumed to consist entirely of fat (Fat), *i.e*. triglycerides, since this is the component of body mass which is soluble in ether. The non-fat dry weight of most insects consists of around 70% protein, 10% structural carbohydrates, 10% other carbohydrates, 6% minerals, with the remainder miscelaneous substances such as nucleic acids [28]. Glycogen and other free carbohydrates, which are important for energy storage in most insects, are essentially absent from tsetse [25] so that protein should contribute around 78% of tsetse RDW. As a first approximation, used for simplicity, this study considers all of the RDW to be protein, *i.e*. polypeptides.

In pregnant female tsetse a significant portion of the bloodmeals are used to produce the milky secretion (milk) of the milk gland which nourishes the offspring *in utero*. This secretion comprises the major gain in body mass of females during pregnancy. This milk consists entirely, by dry weight, of fat and protein [29-31] and is passed on directly to the embryo which has a similar composition. The metabolic pathways of pregnancy therefore involve the conversion of blood to fat and of blood to protein, which are the same pathways as those involved in the manufacture of tsetse body mass.

Tsetse fat acts as an energy reserve which is catabolized for metabolic ATP following two pathways: one pathway, common to all organisms, produces energy for general metabolism and another pathway, specific to tsetse, powers flight by converting alanine to proline and then back [24,32-37].

**Figure 1:**
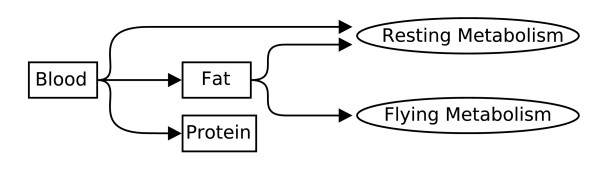
**A schematic overview of the major metabolic pathways in Glossina. **The major metabolic pathways in tsetse involve the conversion of vertebrate blood obtained by feeding into energy (ATP), fat, or protein. Stored fat is catabolized for energy along two pathways, one for general metabolism and another for flight.

The major metabolic pathways of tsetse therefore consist of the conversion of amino acids from blood, into the the energy of the outer bi-phosphate bond in ATP, into body or milk fat, or into body or milk protein, and the conversion of fat into ATP along two seperate pathways, one for resting metabolism and one for flight. These pathways are presented schematically as the five arrows of figure 1.

Crude estimates of the conversion efficiencies of these pathways can be derived from calculations based on the chemical energy content of the end products. Each milligram of blood protein could produce at most between 4.098 *× *10^-4 ^and 5.465 × 10^-4 ^moles of ATP from adenosine diphosphate (ADP), between 0.33 and 0.44 milligrams of Fat, or 1 milligram body protein, since the change in free energy during protein combustion in oxygen is between -3 and -4 kcal/g, the change from the release of the third phosphate of ATP is -7.32 kcal/mol, and the change due to fat combustion is around -9 kcal/g. Each milligram of fat could produce at most 1.230 × 10^-3 ^moles of ATP from ADP. These estimates are high since they do not include the energetic costs and inefficiencies of the conversion process, the costs of disposal of toxic by-products, or the biological costs of digestion and growth. These crude estimates do not provide the amount of respiratory gases consumed or produced in the conversion process and do not distinguish between the two pathways tsetse use to catabolize fat. More detailed estimates require the use of a stoichiometric approach.

The efficiency of conversion along each of these pathways depends, first, on the detailed chemical composition of the reactants, both substrates and products, in these pathways and, second, on the actual sequences of reactions used in each pathway. These two issues are discussed next.

### The detailed chemical composition of the reactants

The exact chemical composition of the reactants involved in these conversions define the end points of the metabolic reaction pathways. This composition constrains the overall yield of the pathway, determines the mass balance of the reactions, and determines the amounts of toxic by-products produced by conversion.

Water, despite forming the major component of the blood food source and of tsetse body tissue, is ignored throughout this analysis because it has no influence on the calculated efficiencies of conversion. Water participates in the biochemical reactions of energy production only as the aqueous reaction medium and as the end product of full catabolism. Since biochemical reactions occur in an aqueous medium, water is never a limiting factor for the reactions. This analysis of the net efficiencies of biochemical conversion can therefore focus entirely on the non-aqueous, 'dry,' components of the tissues.

The biochemical composition of bloodmeal protein is presented in Bursell [22] based on the molar fraction of each amino acids in vertebrate blood. These data are displayed in the second column of table 1. Part of the bloodmeal is not used by tsetse but is egested immediately in the exuvium. The composition of the exuvium is presented in Bursell [22]. The oxygen binding haematin in the bloodmeal is egested without being split into constituent amino acids and both arginine and histidine are absorbed from the gut but then are excreted unmodified, presumably due to their high nitrogen content [22]. Columns 3 and 4 of table 1 show the total nitrogen and carbon mass contained in a pool of one hundred moles of bloodmeal amino acids; both must be accounted for completely in the stoichiometric analysis.

**Table 1:** Amino acids in blood and muscle tissue

**Amino Acids**	**Blood Protein**			**Muscle Protein**		**Milk Protein**
	
	**Bursell, 1965**			**Bursell, 1965**	**Agosin, 1978**	**Cmelik, 1969**
	
	**Amino Acid**	**N**	**C**	**Amino Acid**	**Amino Acid**	**Amino Acid**
	
	**numeric %**	**mol/100 mol**	**mol/100 mol**	**numeric %**	**numeric %**	**numeric %**
**Alanine**	8.3	8.3	24.9	7.4	10.14	5.9
**Arginine**	3.5	*(Excreted)*		7.3	6.08	2.6
**Asparagine**	*(as Aspartic Acid)*			*(below)*	10.66	
**Aspartic Acid**	9.5	9.5	38.0	9.8	*(above)*	5.7
**Cysteine**	2.3	2.3	6.9	0.0	0.00	4.5
**Glutamine**	*(as Glutamic Acid)*			*(below)*	18.70	18.3
**Glutamic Acid**	8.3	8.3	41.5	20.2	*(above)*	
**Glycine**	3.8	3.8	7.6	6.8	5.96	0.9
**Histidine**	6.9	*(Excreted)*		3.3	1.66	0.8
**Isoleucine**	*(as Leucine)*			*(below)*	4.12	
**Leucine**	12.9	12.9	77.4	12.5	10.38	8.5
**Lysine**	9.4	18.8	56.4	9.6	8.59	3.4
**Methionine**	1.2	1.2	6.0	5.7	2.11	w/ cysteine
**Phenylalinine**	6.5	6.5	58.5	5.6	2.97	5.7
**Proline**	4.9	4.9	24.5	4.1	3.03	10.5
**Serine**	4.3	4.3	12.9	*(w/ Glycine)*	4.87	4.3
**Threonine**	5.2	5.2	20.8	3.9	4.25	6.3
**Tryptophan**	0.2	0.4	2.2	0.0	0.00	
**Tyrosine**	3.1	3.1	27.9	3.1	2.38	16.9
**Valine**	9.6	9.6	48.0	*(w/ Methionine)*	4.10	5.7
**Totals**	99.9	99.1	453.5	99.3	100.00	100.0

The amino acid composition of vertebrate blood and tsetse muscle. The composition is reported as number of molecules of each amino acid in 100 moles of blood or muscle amino acids (numeric %). The nitrogen and carbon composition is the number of those atoms contributed by each amino acid in 100 moles of blood amino acids (mol / 100 mol).

The composition of tsetse body fat is estimated in Langley and Pimley [30]. The fatty acid composition of tsetse fat consists approximately of 40% palmitic acid (16 carbons:0 double bonds), 30% palmitolic acid (16:1) and 20% oleic acid (18:1) [30]. For this analysis, all fat is assumed to consist of triesters of palmitic acid. This approximation should only lead to a minor error in the estimated yield since the sixteen carbon chain represents a reasonable average chain length [22], because the energetic difference between equal masses of different fatty acids are minor, and since the energtic cost of double bond formation is minor.

The composition of tsetse muscle protein is presented in Bursell [22] and can be recalculated from Agosin [38]. Those results are repeated in columns 5 and 6 of table 1. This study considers the amino acid composition of tsetse flight muscle protein to be identical to the composition of vertebrate blood both based on the similarity of the second, fifth, and sixth columns of table 1 and because the differences between the two estimates of tsetse muscle protein composition are on the scale of their differences to vertebrate blood. A more accurate analysis would require a series of dedicated experiments providing estimates of the composition of these tissues and assessing the stability of this composition over time. Based on the simplification used here, the formation of tsetse muscle protein is considered to require a bulk transfer of amino acids obtained from the bloodmeal along with the energetic cost of peptide bond formation.

The composition of the fat produced in the milky secretion of females and incorporated into the developing embryo was analyzed by Langley and Pimley [31]. They found that the fat in milk consists of approximately 65% palmitic acid (16:0), 27% palmitolic (16:1), 7% linoleic acid (18:2), and 2% myristic acid (14:0). As stated above, this analysis considers all fat to be triesters of palmitic acid, an assumption which should not lead to major errors in the final estimated efficiencies of conversion.

The composition of the protein in tsetse milk is presented in an article by Cmelik *et al*. [29] and repeated here in column 7 of table 1. The amino acid composition of the milk differs from the composition of blood and muscle principally in the elevated fractions of proline, glutamate, and tyrosine [39]. This analysis was unable to integrate this difference in amino acid compositions; instead, the composition of milk protein is assumed identical to the composition of the protein in vertebrate blood and in tsetse muscle. This simplification should lead to an overestimate of the yield of milk mass from a given amount of blood because the three amino acids present in higher proportions have a higher energy content than the average amino acid so the production of these amino acids would require the conversion of part of the blood to energy. However, the simplifying assumption is required by the limitations in the methodology of the study and in the data used for this analysis. A more refined approach is left to future studies.

Tsetse dispose of the excess nitrogen in the bloodmeal by egesting haematin, by excreting arginine and histidine, and by forming uric acid [22,40]. There is no published evidence of the excretion, by tsetse, of ammonium so all disposed nitrogen is assumed to be excreted as uric acid. The formation of uric acid requires both carbon for the molecule's rings and ATP to drive the reaction. These costs of nitrogen disposal must be included in any realistic estimate of the net conversion yields along each of the major metabolic pathways.

In this paper, the five major metabolic pathways presented in figure 1 have been simplified to the following conversions. Tsetse food is assumed to consist exclusively of amino acids in the proportions presented by Bursell [22]. This pool of amino acids is converted into ATP, into triesters of palmitic acid, or into protein with identical amino acid composition to the blood. The triesters of palmitic acid are themselves catabolized to form ATP along either of two metabolic pathways.

### The reaction sequence along each pathway

The sequence of reactions used by tsetse for the conversions described by figure 1 can be obtained by linking general descriptions of the metabolic pathways used by tsetse, obtained from the tsetse literature, with specific descriptions of the biochemical reactions which occur in these pathways, obtained from the biochemical literature.

The metabolic pathways used by tsetse combine standard pathways used by most organisms with pathways specific to tsetse. The formation, from amino acids, of ATP and of fat proceed through standard pathways, respectively, the Krebs cycle followed by the electron transport chain and the triglyceride synthesis pathways. The pathway used by tsetse to form uric acid proceeds via glycine [22,24]. Tsetse use both the standard pathway of fat catabolism through the Krebs cycle and use a special pathway for flight energy which combines the common path used to convert fat to proline [24,33,35,41] with the common path used to convert proline to ATP [24,34,36,37] while alanine acts as the transamination reactant [42].

The specific reactions involved in each step of the conversion pathways are similar for most organisms and have been established by biochemists and presented in the biochemical literature. The book *Biochemical Pathways *by Michal [12] provides an integrated presentation of the biochemical reactions which occur in living organisms. This book describes every reaction involved in each of the metabolic pathways presented in figure 1.

Biologically realistic calculations of the proportions of conversion between substrates (blood or fat) and products (ATP, fat, or protein) in the five pathways presented in figure 1 must include the energetic costs incurred in the reactions of conversion and the costs of disposal of the toxic by-products of each pathway. ATP is required for the formation of both fat and protein and for certain steps in most reaction pathways. To generate the required ATP, a proportional quantity of the reaction substrate must be catabolized. The amino acid diet of tsetse is high in nitrogen whose accumulation could potentially reach toxic levels. The formation of uric acid to excrete this excess nitrogen requires both energy and carbon which must be derived from some of the amino acids in the bloodmeal.

**Figure 2:**
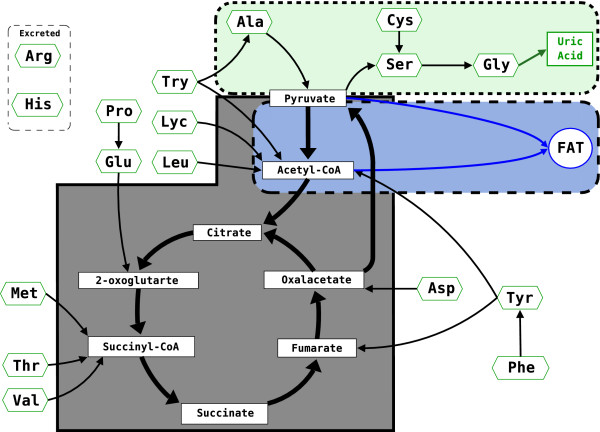
**The biochemical pathways involved in digestive conversion in tsetse. **The biochemical conversion pathways used by tsetse. Amino acids from the bloodmeal (hexagons) enter the reactions of conversion as Krebs cycle constituents (rectangles), as shown by the arrows. The dark area (grey) includes the Krebs cycle, the lighter area (blue) outlines the pathways of fat creation and catabolism, and the lightest area (green) shows the reactions of uric acid formation.

An overview of the biochemical conversions considered here is presented in figure 2. The dark area (gray) includes the Krebs cycle, the lighter area (blue) outlines the triglyceride (fat) metabolic pathway, and the lightest shaded area (green) includes the elements involved in the formation of uric acid. The hexagonal labels denote the amino acids and the rectangular labels identify Krebs cycle products. The entry points of amino acid into the Krebs cycle are taken from McCabe and Bursell [41](see figure 3) and Michal [12](see figure 3.8-1). Threonine (Thr), capable of entering the cycle either as Acetyl-CoA or as Succinyl-CoA, is assigned entirely to Succinyl-CoA following McCabe and Bursell [41]. The estimates of the energetic yields of Krebs cycle constituents are presented in table 2. These are used to obtain the estimates of the energetic yields of amino acid catabolism given in table 3.

**Table 2:** Krebs cycle yields and gas exchange

**Substrate**	**ATP Created**	**CO_2 _Released**	**O_2 _Consumed**
**Pyruvate**	12.5	3	2.5
**Acetyl-CoA**	10	2	2
**2-oxoglutarate**	21	5	4
**Succinyl-CoA**	18.5	4	3.5
**Fumarate**	16	4	3
**Oxalacetate**	13.5	4	2.5

The gross energy yield and gas exchange in the catabolism of Krebs cycle elements in atoms per amino acid converted.

**Table 3:** Amino acid catabolism

**Amino Acid Substrate**	**Intermediate Product**	**ATP Created**	**CO2 Released**	**O2 Consumed**
**Alanine**	Pyruvate	12.5	3	2.5
**Arginine**	*(Excreted)*			
**Aspartate**	Oxalacetate	13.5	4	2.5
**Cysteine**	Pyruvate	12.5	3	2.5
**Glutamate**	2-oxoglutarate	21	5	4
**Glycine**	1/2 Pyruvate	7.5	2	1.5
**Histidine**	*(Excreted)*			
**Leucine**	3 Acetyl-CoA	33	6	7
**Lysine**	2 Acetyl-CoA	29	6	6
**Methionine**	Succinyl-CoA	18	5	4
**Phenylalinine**	Fumarate & 2 Acetyl-CoA	36	9	10
**Proline**	2-oxoglutarate	26	5	5
**Serine**	Pyruvate	12.5	3	2.5
**Threonine**	Succinyl-CoA	20	4	4
**Tryptophan**	2 Acetyl-CoA & Pyruvate	22.5	11	10.5
**Tyrosine**	Fumarate & 2 Acetyl-CoA	36	9	9
**Valine**	Succinyl-CoA	25	5	5

The gross energy yield and gas exchange in amino acid catabolism based on the estimated energy yields of Krebs cycle elements given in table 2, in atoms per amino acid converted.

### Overview of this analysis

The results section presents the estimated yield, uric acid production, and gas exchange in each of the five major metabolic pathways. First, the analysis examines the pathway of conversion of blood into the energy in the outer bi-phosphate bond of ATP, requiring an estimate of the concomitant cost of uric acid formation. Next, conversion estimate of blood to protein is calculated as a bulk transfer of blood amino acids to protein coupled with the cost of protein formation. Third, the conversion of blood to fat is analyzed. Finally, the last two sections consider the catabolic breakdown of fat for the formation of ATP from ADP, first, following the pathway used for resting metabolism, and then, following the pathway used for flight.

The discussion section evaluates the yield estimates by comparing them to empirical measurements described in the literature, then assesses the results, examines the assumptions, and considers the validity of the approach.

## Results

The conversion efficiency of each of the five pathways of figure 1 is considered separately below. The spreadsheet file used to calculate the estimates is included with the manuscript both in the open Gnumeric file format (a GNU Zip compressed XML file) [see Additional file 1] and in the unpublished Microsoft Excel file format [see Additional file 2].

### Blood catabolism for ATP formation

The first major metabolic pathway involves the conversion of the amino acids obtained from the protein in a bloodmeal into the high energy bonds in ATP used to power cellular processes and muscular motion. All adult tsetse use part of their bloodmeals for this purpose. The net estimate of the yield of this conversion pathway must incorporate the costs of uric acid formation required to excrete all of the nitrogen in the bloodmeal.

The proportion of conversion of blood to ATP is calculated starting, for convenience, with a pool of blood which consists of 100 moles of amino acids in the molar proportions of human blood presented in [22] and repeated in the second column of table 1. The starting pool of amino acids, after accounting for the excretion of arginine and histidine and for the amino acids which contain several nitrogen atoms, contains 99.1 moles of nitrogen and 453.5 moles of carbon, as presented in the totals of the third and fourth columns of table 1.

When bloodmeal amino acids are converted to ATP, all of the absorbed nitrogen is excreted as uric acid [22]. Uric acid formation requires the use of a glycine carbon backbone, the input of carbon from both carbon dioxide and formate, the inclusion of nitrogen from the transamination of glutamine to glutamate and the input of energy. This analysis assigns all of the glycine, serine, and cysteine and part of the alanine in the blood meal to form the glycine precursors to uric acid. These four amino acids are present in sufficient concentrations in the bloodmeal to provide all the glycine required to form enough uric acid to dispose of all the absorbed nitrogen. Both the carbon dioxide and the energy required in the manufacture of this uric acid can be obtained from the catabolic degradation of some of the amino acids in the original pool.

The overall costs of uric acid formation are calculated as conversions of alanine and cysteine to serine, serine to glycine and glycine to uric acid based on the reaction sequences in Michal [12], with the parentheses in this paragraph indicating the figure from that work documenting the reactions. The conversions of alanine to pyruvate (4.2-1) and cysteine to pyruvate (4.5-3) are followed by the conversion of pyruvate to serine (4.4-1). Serine is converted to two glycine molecules (4.4-3) and, finally, the glycine is converted to uric acid (8.1-2 and 8.1-4). The overall costs of uric acid formation from each amino acid precursor are as follows:

1 Alanine + 7 CO_2 _+ 1 O_2 _+ 23 ATP → 2 Uric Acid

1 Cysteine + 7 CO_2 _+ 1 O_2 _+ 23 ATP → 2 Uric Acid

1 Glycine + 3 CO_2 _+ 0.5 O_2 _+ 11.5 ATP → 1 Uric Acid

1 Serine + 7 CO_2 _+ 0.5 O_2 _+ 25.5 ATP → 2 Uric Acid

The numbers of ATP molecules required are higher than the results presented for avian biochemistry by Stevens [43](see page 75) because this analysis includes the two NADH molecules needed to recreate formate for the activation of N^10^-Formyltetrahydrofolate [12](see figure 9.6-1) and includes a NADH molecule released during the conversion of Inosine-5'-Phosphate to Xanthosine-5'-Phosphate [12](see figure 8.1-4).

Taking 3.89 moles of alanine, 2.30 moles cysteine, and 4.30 moles serine, each of which produce twice the amount of uric acid, and 3.80 moles glycine, 24.78 moles of uric acid are produced. Since each uric acid molecule holds four nitrogen atoms, this quantity of uric acid accounts for the 99.1 moles of nitrogen in the original pool of 100 moles of amino acids (the total of the third column of table 1). This process requires approximately 296 moles of ATP, 85 moles of carbon dioxide, and 10 moles of oxygen.

The remaining amino acid molecules can be fully catabolised through the Krebs cycle to form high energy phosphate bonds in ATP. The reactions of catabolism are described in the text and figures of Michal [12], as follows. The conversions of each amino acid into pyruvate, acetyl-CoA, or Krebs cycle elements are described in the figures of chapter 4 which covers amino acid biochemistry. Additionally, the conversions of pyruvate and acetyl-CoA to elements in the Krebs cycle are presented in figure 3.3-1, the Krebs cycle is shown in figure 3.8-2, and the ATP yields of the oxidation of both NADH and FADH_2 _are given in section 3.8.3. Using this information, the calculated yields of catabolism are presented in the tables of this paper, table 2 for the Krebs cycle products and table 3 for the amino acids themselves. This conversion requires oxygen for the reduction of NADH and FADH_2_. The carbon contained in the amino acids is released as carbon dioxide. The sulfur in cysteine and methionine is simply ignored; it is likely excreted in the pigmented fraction of the exuvium [22].

The yield of converting the amino acids remaining from the original pool of 100 moles of bloodmeal amino acids after accounting for the costs of uric acid formation are presented in table 4. The catabolic degradation of the remaining amino acids generates approximately 1900 moles of ATP and 414 moles of carbon dioxide while using about 408 moles of oxygen. The 414 moles of carbon dioxide released in the catabolic breakdown of these amino acids, when combined with the carbon contained in the amino acids used as precursors to uric acid (3 per mole of alanine, cysteine, and serine, 2 per mole of glycine), account for all of the 453.5 moles of carbon in the original pool of amino acids.

**Table 4:** Yield of available amino acids

**Amino Acid**	**Remaining ** **mol**	**ATP Gain** **mol**	**CO_2 _released** **mol**	**O_2 _used** **mol**
**Alanine**	4.41	55.16	13.24	11.03
**Aspartic Acid**	9.50	128.25	38.00	23.75
**Glutamic Acid**	8.30	174.30	41.50	33.20
**Leucine**	12.90	425.70	77.40	90.30
**Lysine**	9.40	272.60	56.40	56.40
**Methionine**	1.20	21.60	6.00	4.80
**Phenylalinine**	6.50	234.00	58.50	65.00
**Proline**	4.90	127.40	24.50	24.50
**Threonine**	5.20	104.00	20.80	20.80
**Tryptophan**	0.20	4.50	2.20	2.10
**Tyrosine**	3.10	111.60	27.90	27.90
**Valine**	9.60	240.00	48.00	48.00
**Totals**		1899.11	414.44	407.78

The results of the catabolic conversion of the amino acids remaining from a pool of 100 moles of blood derived amino acids after accounting for the total disposal of the nitrogen through uric acid formation.

The overall catabolism of the bloodmeal for energetic production is simply the combination of the uric acid disposal pathway and the catabolism of the remaining amino acids. The overall conversion of 100 moles of blood derived amino acids results in 25 moles of uric acid, 1600 moles of ATP, and 330 moles of carbon dioxide while using 420 moles of oxygen. The full catabolism of the blood meal has a calculated respiratory quotient of 0.79, close to the 0.8 value usually used for protein.

These numbers become more meaningful when considered in terms of mass. For convenience we can start with 1.0 mg of dry blood. Dry blood is approximately 20% of the wet weight of blood [22,44] so 1.0 mg dry blood would correspond to 5.0 mg of wet blood meal. Of the 1.0 mg dry blood, approximately 0.88 mg is non-haematin protein available for digestion [22]. In the separation of protein into amino acids, water of hydration is absorbed giving the amino acids 1.14 times the mass of the protein [22] so the resulting mass of amino acids is once again around 1.0 mg. The amino acids in this pool have a proportional average mass of 131.51 g per mole calculated from the proportions of amino acids in blood given by Bursell [22] and the molecular mass of each amino acid. The milligram pool of amino acids therefore contains 7.61 × 10^-6 ^moles of amino acids. Using this number of molecules of blood derived amino acids and the yields established above, a 1.0 mg dry bloodmeal yields 1.22 × 10^-4 ^moles of ATP while requiring the formation of 0.32 mg uric acid. Based on the ideal gas law at standard temperature (25°C) and pressure (1 atmosphere), 1.0 mg of dry blood requires 782 mm^3 ^oxygen and yields 615 mm^3 ^of carbon dioxide when degraded entirely to create ATP.

### Blood conversion to protein

The second major metabolic pathway involves the conversion of blood into protein. Adult flies which have just emerged from the puparial stage create protein to increase their flight musculature [45,46]. Mature pregnant females form protein as part of the milk which feeds the developing offspring. Based on the assumption of equivalence between the amino acid composition of blood, of muscle, and of milk protein, the formation of muscle or milk protein consists simply of the direct transfer of a portion of the bloodmeal amino acids coupled with the use of 4 ATP per amino acid [12](see page 135) to form the protein chain. This estimated energetic cost of protein formation ignores the possible energetic recovery from the pyrophosphate released in the reaction linking each amino acid to the transfer RNA molecule [47](see page 963). The other costs of protein manufacture, including all of the cellular processes required to develop and maintain the protein synthesis infrastructure, are also ignored so that this analysis underestimates the costs and therefore overestimates the amount of protein generated from a given bloodmeal.

Starting again with 100 moles of original amino acids, the conversion of this entire pool of amino acids to protein requires 100 moles of peptide bonds or 400 moles of ATP. By expanding the original pool with an additional 25 moles of amino acids, the 400 moles of ATP required can be obtained since the previous section showed that 100 moles of amino acids yield 1600 moles of ATP. Therefore the pool of 100 moles of amino acids which are used for the protein biomass must be combined with the 25 moles of amino acids used to generate energy thereby requiring a total of 125 moles of amino acids from the blood to create protein containing 100 moles of amino acids. Equivalently, 100 moles of blood amino acids can be partitioned into 80 moles to make protein while burning the remaining 20 moles for metabolic energy and uric acid formation. This process would generate 1/5^*th *^of the uric acid and carbon dioxide from the previous section and require 1/5^*th *^of the oxygen.

In mass terms, 1 mg of dry blood can be used to create 0.80 mg of muscle or milk protein requiring the formation and excretion of 0.06 mg uric acid, the inhalation of 156 mm^3 ^of oxygen, and the exhalation of 123 mm^3 ^of carbon dioxide.

### Blood conversion to fat

The third major metabolic pathway in tsetse involves the use of the bloodmeal to create triglycerides in the fat body. All adult flies must convert part of the bloodmeal to energetic reserves and pregnant female flies will use part of the blood allocated to reproduction to create the triglycerides in the secreted milk.

Starting again with an original pool of 100 moles of blood derived amino acids, a first allocation will be to the formation of uric acid. Fat does not contain nitrogen so all of the nitrogen in the bloodmeal must be formed into uric acid and excreted before fat formation. This process is identical to the excretion of nitrogen involved in the pathway converting blood to ATP so the numbers presented in that section work for this pathway as well.

The remaining amino acids provide both the carbon precursors of the fat molecules and the energy required for synthesis. The reactions are taken from Michal [12] and listed here in parenthesis. The synthesis of triesters of palmitic acid requires the combination of one pyruvate molecule to make the glycerol head (figure 3.1-1) with 24 acetyl-CoA molecules to make the fatty acid chains (section 6.1-4). Linking the acetyl molecules into fatty acid chains and then to the glycerol head (section 6.2-1) requires 137 ATP molecules to drive the synthesis. The amino acids remaining after accounting for uric acid formation are therefore assigned proportionally to pyruvate, acetyl-CoA, and ATP. Carbon dioxide is released and oxygen consumed only in the process of catabolic degradation for ATP production.

The balanced use of the amino acids in the bloodmeal after uric acid formation comes from the creation of 4.08 moles of fat, necessitating 4.08 moles of pyruvate, 98 moles of acetyl-CoA (4.08 × 24), and 560 moles of ATP (4.08 × 137). The required pyruvate can be obtained by combining 3.88 moles of alanine, the amount remaining after uric acid formation, with 0.2 moles of pyruvate generated from tryptophan degradation. The 98 moles of acetyl-CoA, shown in table 5, column 3, can be generated with the quantities of amino acids presented in table 5, column 2. The 560 moles of ATP can be generated through the catabolic conversion of the amino acids remaining after accounting for the amino acids used to form uric acid, pyruvate, and acetyl-CoA (shown in table 6). This ATP is the sum of the 654 moles from amino acid catabolism (shown as the total of the third column of table 6) plus the 200 moles arising from the creation of acetyl-CoA (shown as table 5, column 4), minus the 296 moles incurred in the creation of uric acid.

**Table 5:** Amino acids used to form acetyl-CoA

**Amino Acid**	**To Acetyl-CoA ** **mol**	**Acetyl-CoA ** **mol**	**ATP Gain** **mol**	**CO_2 _released** **mol**	**0_2 _used** **mol**
**Alanine**	0.50	0.50	1.25	0.50	0.25
**Aspartic Acid**	9.50	9.50	33.25	19.00	4.75
**Glutamic Acid**	3.04	3.04	33.42	9.11	6.08
**Leucine**	12.90	38.70	38.70	0.00	12.90
**Lysine**	9.40	18.80	84.60	18.80	18.80
**(Phenylalinine)**	6.50	13.00	*(Added to energetic catabolism.)*		
**Threonine**	5.20	7.80	9.10	5.20	3.90
**Tryptophan**	0.20	0.40	0.80	0.80	0.80
**(Tyrosine)**	3.10	6.20	*(Added to energetic catabolism.)*		
**Totals**		97.94	200.32	52.61	46.68

The amino acids from the bloodmeal assigned in this analysis to the synthesis of acetyl-CoA for the fatty acid chains of the fat molecule. Phenylalinine and tyrosine yield a mixture of Krebs cycle products as shown in figure 1 and only the fractions yielding acetyl-CoA are considered here, the other fractions are considered in the synthesis of ATP presented below. Tryptophan is catabolized into both the pyruvate described above and an acetyl-CoA fraction included in this table.

**Table 6:** Amino acids used for ATP formation

**Amino Acid**	**To ATP** **mol**	**ATP Gain** **mol**	**CO2 released** **mol**	**O2 used** **mol**
**Alanine**	0.03	0.41	0.10	0.08
**Glutamic Acid**	5.26	110.50	26.31	21.05
**Methionine**	1.20	21.60	6.00	4.80
**(Phenylalinine)**	6.50	104.00	32.50	39.00
**Proline**	4.90	127.40	24.50	24.50
**(Tyrosine)**	3.10	49.60	15.50	15.50
**Valine**	9.60	240.00	48.00	48.00
**Totals**		653.51	152.91	152.93

The amino acids catabolised to fuel triglyceride assembly. Only the parts of phenylalinine and tyrosine not used in the creation of pyruvate and acetyl-CoA are consumed to generate ATP.

The net effect of the conversion of 100 moles of blood amino acids into fat is the creation, as before, of 25 moles of uric acid, the creation of 4.1 moles of fat, the release of 120 moles of carbon dioxide, and the consumption of 210 moles of oxygen.

In mass terms, 1 mg of dry blood yields 0.25 mg fat, 0.32 mg uric acid, 224 mm^3 ^carbon dioxide, and requires 391 mm^3 ^oxygen.

### Fat catabolism

The two remaining major metabolic pathways both involve the catabolic degradation of fat to obtain ATP. The first pathway is used by tsetse during all stages of life. This pathway proceeds directly through the Krebs cycle. The second pathway is used by adult tsetse to provide energy to flight muscles and involves the transformation of alanine to proline and back. These two pathways are considered below.

The first pathway of triglyceride degradation involves, first, the split of the triester into the glycerol head and the three fatty acid chains [12](see figure 6.2-1), then, the sequential split of an acetyl group from each fatty acid [12](see figure 6.1-9), and, finally, the degradation of both the glycerol and acetyl molecules via the Krebs cycle [12](see figures 3.1-1, 3.3-1, and 3.8-2). The conversion of one triglyceride made of three palmitic acid chains proceeds as follows

1 Triglyceride + 8 ATP → 25 Acetyl-CoA + 24 NADH + 21 FADH_2 _+ 1 CO_2_

or

1 Triglyceride + 72.5 O_2 _→ 333.5 ATP + 51 CO_2_

for each triester of palmitic acid.

The second pathway of fat catabolism starts with the degradation of the triglyceride as above to acetyl-CoA. The acetyl-CoA molecules are then used to convert alanine through the Krebs cycle to proline. This proline is transported to the flight muscles and degraded, through the other reactions of the Krebs cycle, back to alanine during flight. The formation of proline, based on the pathway presented in McCabe and Bursell [33](see figure 3) and the reactions in Michal [12], is:

1 Alanine + 1 Acetyl-CoA + 2 NADH + 2 ATP → 1 Proline

This equation differs from that presented in Bursell [35](see figure 3) by accounting for the molecule of ATP required to activate E-Biotin with the carbon dioxide molecule as part of the pyruvate carboxylase pathway [12](see figure 3.3-1) and by accounting for a slight difference between the modern understanding of the Krebs cycle and the interpretation of Bursell's. In the flight muscle sarcosome, the proline is degraded back to alanine yielding:

1 Proline + 3 O_2_→ 1 Alanine + 2 CO_2 _+ 15 ATP

according to the pathway presented in McCabe and Bursell [41](see figure 3) and the reactions in Michal [12]. This equation differs from that presented by Bursell [37] but only in one atom of oxygen and one of ATP. When these two equations are combined, the degradation of each acetyl radical derived from the fat molecule results in the overall equation:

1 Acetyl-CoA + 2 O_2_→ 2 CO_2 _+ 8 ATP

This pathway generates two fewer ATP per acetyl radical degraded than does degradation through the Krebs cycle. The amount of oxygen released has been calculated based on offsetting the NADH molecules needed to create proline with those generated in the degradation of proline. Since these processes may occur at different times, this estimate of oxygen use only works as a long term average.

Generating energy through this pathway, using the modern interpretation, would result in the overall degradation of one triglyceride molecule as follows.

1 Triglyceride + 72.5 O_2_→ 283.5 ATP + 51 CO_2_

This pathway provides less energy than the general pathway of oxidation presented above, entirely due to the differing yields in the degradation of the acetyl radicals.

Converting these numbers to mass terms, 1 mg of tsetse fat can be used to generate 4.13 × 10^-4 ^moles of ATP for the first pathway or 3.51 × 10^-4 ^moles for the second. Both pathways release 1550 mm^3 ^of carbon dioxide and, despite differing in energetic yield, both require 2200 mm^3 ^of oxygen. The calculated respiratory quotient of fat catabolism is 0.70, which is the value generally used for fats and the value presented by Michal [12](see page 408).

### Overall results

The yields in each of the five major metabolic pathways of figure 1 are presented in table 7. The table includes estimates of the proportions of conversion of blood to ATP, fat, and protein and of fat to ATP through the two pathways used by tsetse. The table also includes the proportion of uric acid formed to excrete excess nitrogen and the volumes of carbon dioxide released and oxygen consumed in each conversion process.

**Table 7:** Overall Results

**Substrate**	**Product**		**Uric Acid ** (mg)	**CO_2 _** (mm^3^)	**O_2 _** (mm^3^)
**Blood **(1 mg dry)	ATP	1.221 × 10^-4 ^mol	0.3199	614.5	782.1
	Fat	0.2458 mg	0.3199	223.5	391.1
	Protein	0.8008 mg	0.06398	122.9	156.4
**Fat **(1 mg)	ATP	4.131 × 10^-4 ^mol	-	1545	2197
	ATP (via Proline)	3.512 × 10^-4 ^mol	-	1545	2197

The calculated yield estimates of the five major metabolic pathways in tsetse and the associated uric acid formed, carbon dioxide released and oxygen used. ATP indicates the additional formation of one phosphate to phosphate bond, Fat refers to triesters of palmitic acid, and Protein indicates polypeptide chains. Volumes were calculated assuming a standard temperature of 25°C and pressure of 1 atmosphere. The accuracy of these numbers exists only to report calculated results; these numbers are probably only accurate to the first significant figure. The estimated volume of oxygen released during fat anabolism is probably too high as explained in the discussion.

## Discussion

The estimates presented in table 7 can be evaluated by comparison against experimental results.

Bursell [22] estimated the amount of uric acid excreted following a bloodmeal to be directly proportional to the size of the meal. He estimated that a 1 mg dry mass bloodmeal would lead to the formation of 0.280 mg uric acid for the first meal and 0.342 mg in subsequent meals. These measurements compare favorably with the calculated values presented here of 0.32 mg uric acid per mg dry bloodmeal for the production of either ATP or fat and of 0.06 mg uric acid per mg dry bloodmeal in the production of protein. Flies feeding for the first time are investing much of their bloodmeals into the formation of flight musculature [45,46] so the value measured by Bursell should be due to the use of the bloodmeal for both conversion to ATP and manufacture of protein. The measured value for the first bloodmeal can be explained from the calculated values by assuming 15.4% of the first bloodmeal is used to make protein and the rest is used to make energy or fat. The measured value in later meals, while greater than the calculated values for the production of energy and fat, is nonetheless close to the value calculated here. The calculated results account for all of the nitrogen in the bloodmeal so that it is not necessary to consider the possibility of nitrogen excretion through ammonium.

Rajagopal and Bursell [48] measured the rate of respiration in resting tsetse in the days after feeding. They estimate a three day total respiration of 2184 mm^3 ^oxygen following a bloodmeal of 25 mg wet mass. Using the calculated rates presented here, the same bloodmeal would consume approximately 3900 mm^3 ^of oxygen if all the blood were converted to energy, 2000 mm^3 ^of oxygen if the blood were made into fat, and 800 mm^3 ^of oxygen if the blood were made into protein. Since these numbers bracket the measured result, it is possible to explain the measurement as a combination of the calculated values, demonstrating the plausibility of the estimates presented here.

Puparia of tsetse do not feed but depend on accumulated energetic reserves. Puparia of *Glossina morsitans orientalis *Vanderplank consume roughly 4200 mm^3 ^oxygen total during this phase of their lifecycle [49] and use approximately 2.0 mg fat [50]. From the calculations presented here, the conversion of 2.0 mg fat to energy would require 4400 mm^3 ^oxygen which falls within 5% of the measured result, again validating these estimates.

These comparisons indicate that the yield proportions calculated in this paper appear plausible as initial estimates despite the disparate sources of analytic data, the many assumptions and simplifications required by the analysis, and the issues with the methodological approach of this work. The stoichiometric approach seems to account for the bulk of experimental observation based on its mechanistic explanation of chemical reaction yields.

While the overall results appear to be plausible, certain of the assumptions on which the yield calculations were based appear especially problematic and should be revisted. The assumption that all metabolic energy can be treated as equivalent to ATP correctly quantifies the energetic costs but can lead to a significant over-estimate of the oxygen required by a conversion pathway. This assumption implicitly assumes that all of the energy obtained from NADH and FADH_2 _involves the generation of ATP through the electron transport chain with the concomitant use of oxygen. However, some of the conversion pathways involve reactions which use NADH and FADH_2 _directly. The assumption has a trivial effect on the estimate of the oxygen use in the conversion of blood protein into ATP. Part of the energetic cost of uric acid formation can be met directly by NADH, accounting for 2.5 of the 11.5 ATP equivalents per uric acid molecule. Since this accounts for roughly 22% of this energetic cost, the estimate of required oxygen for this path should be lowered by this amount. However, the effect of the assumption on the estimate of the overall oxygen requirement is insignificant, leading to an increase of two moles out of the 420 moles total oxygen use estimate, an effect of less than 1%. The assumption does have a significant effect on the estimate of oxygen use in the conversion of blood protein into fat. Partly this is due to an overestimate of oxygen use in the formation of uric acid, but that effect is tiny. Predominantly, the effect comes from the energetic cost of triglyceride formation in which, of the 137 ATP equivalents required per triglyceride, 110 can be met with NADH directly. The overall estimate of oxygen use should therefore be lowered by around 77% to approximately 90 mm^3^, leading to respiratory quotient of around 2.5. The assumption should not have any significant effect on the estimate of oxygen use during conversion of blood into body protein production. A trivially small error does arise due to the assumption because protein formation requires ATP for peptide bond formation and ATP production overestimates oxygen use by around 1% due to the error in uric acid formation. The assumption should not have any effect on the estimates of oxygen use during fat catabolism to ATP along either pathway.

The assumption that tsetse body mass can be treated simply as a combination of water, palmitic acid, and muscle protein ignores the contributions of carbohydrates, such as chitin, of nucleic acids, such as transcription RNA, and of other non-proteinaceous body mass components but this omission should not lead to any major error. A more accurate representation of the body fat fraction of tsetse mass should not greatly alter the results obtained here since the energetic difference in the manufacture of equal masses of different fats is relatively small. The assumption that RDW is composed entirely of muscular protein will affect the results of the analysis depending on the relative proportions of these other tissues, on differences in the costs and efficiencies of the reactions of conversion, and on the differences in the energy content of these tissues compared to protein. Structural carbohydrates account for only around 10% of typical insect body mass [28], have a similar energy content to protein, and differ in formation costs primarily due to uric acid formation which requires only 10% by mass of the original pool of amino acids. The treatment of chitin as protein should therefore only have a small effect on the yield estimate. Non-structural carbohydrates do not form a significant part of tsetse body mass [25]. Nucleic acids account for only around 4% of typical insect body mass [28]. The assumption that RDW can be treated as comprised entirely of protein therefore appears to lead only to a small error. Both the assumption of the equivalence of the amino acid composition of vertebrate blood, tsetse muscle, and female milk and the simplification of protein manufacturing costs to peptide bond formation lead to overestimates in the amount of protein produced from the blood. Preliminary analysis suggests the correct yield might be as low as 0.56 mg milk protein per milligram blood instead of the 0.80 mg reported here. However, accurate estimates incorporating differences in amino acid composition require both better data to demonstrate the consistency of the amino acid composition in different species, samples, locations, and time periods and better methods of calculation able to obtain a true maximum yield for the pathways described. This analysis ignores the pathway of carbohydrate synthesis and catabolism which could be an important, albeit temporary, metabolic pathway. Tsetse do not use carbohydrates to store energy, as was stated earlier, but might nonetheless use the pathway in their metabolism. The cyclic creation and use of carbohydrates from Krebs cycle precursors requires no oxygen, releases no carbon dioxide, and only consumes small amounts of energy. Ignoring this pathway, even if it were used, should therefore not have any significant impact on the calculated yields. These assumptions were necessary in this paper to obtain the estimates of table 7 but are not inherent to the biochemical approach. Future studies could therefore refine these estimates.

The yield estimates presented in table 7, while they improve on the current understanding, could nonetheless be improved. The biochemical approach leads to estimates which are significantly lower than the crude estimates which were presented in the background section and which were based on the energy content of the tissues. The yield estimates of ATP in the catabolic pathways are between 22% and 30% of the crude estimate. The yield estimate of fat is between 56% and 74% of the crude estimates, while the yield estimate of protein is 80% of the crude estimate. These modifications are due to considering the energetic costs of the transformation, to considering the biomass and energetic costs of uric acid formation for the disposal of nitrogen, and to an approach which incorporates the inefficiencies of the biochemical reactions involved in conversion. However, all of the biological costs of digestion, growth, and transcription are still ignored. Because both nitrogen and carbon are tracked explicitly in the stoichiometric equations, the estimated amounts of uric acid formed and of carbon dioxide released should be essentially exact. The estimated amount of oxygen required is still inaccurate. The oxygen requirement depends primarily on the amount of ATP formed through the electron transport chain. As was discussed above, the treatment of all energy as ATP equivalents leads to a significant overestimate of the oxygen requirement in the conversion of blood protein to body fat.

The biochemical approach and the calculation strategy used in this paper succeed in providing working numbers for future research but suffer from a number of limitations. The derivation of tissue composition from data obtained by disparate studies leads to an inconsistent basis for the rest of the analysis and lacks detail such as the variation in these tissue compositions between species and over time. This could be improved through direct experimental analysis. The derivation of the reaction sequence from the figures of *Biochemical Atlas *[12] does not guarantee obtaining either the real or an optimal pathway. This could be improved using an approach based on stoichiometric matrices [51,52] which would include multiple alternative pathways and might allow numerical determination of the optimum. The determination of the stoichiometric balance of each reaction from graphical representations is prone to error. An alternative derivation based on direct access to the proteinomic databases could avoid this error. The use of a spreadsheet to perform the calculation was problematic and could be improved through the use of an approach based on stoichiometric matrices. In the future, a better approach than that used here would be to improve and use the informatic tools available for this type of study. Such tools would use complete databases of known metabolic reactions to construct stoichiometric matrices which include the reactions along all plausible pathways. The tools would then solve the matrices using computer algorithms to automatically calculate maximum yields of products and byproducts. Several long term projects seem to be working on such an informatic platform but face numerous difficulties in the integration of biochemical data, in the development of graph traversal and optimization algorithms, and in the creation of flexible interfaces for researchers to use.

Since the estimates presented in this paper appear plausible as a first approximation, the impact of these yield ratios can be briefly considered by comparing the efficiency of energy generation along the three ATP generating pathways. The pathway involving conversion of the bloodmeal to fat and then to ATP is 83% as efficient as the pathway of direct conversion of the bloodmeal to ATP. The pathway used to provide the metabolic ATP during flight is 70% as efficient as the simple conversion of blood to ATP and 86% as efficient as the pathway of fat catabolism directly through the Krebs cycle. It is interesting to note how little energy is lost by the indirect transformations.

## Conclusion

This approach to the analysis of the metabolic efficiency in tsetse succeeds in obtaining the all desired estimates, in improving on the calorimetric approach, and in obtaining estimates which appear reasonable when compared to experimental results. The stoichiometric approach was able to derive yield estimates for each of the major metabolic pathways in tsetse, as described by figure 1, and to include estimates of the uric acid which must be excreted during each of these conversions, of the carbon dioxide released by the conversion, and of the oxygen required. These estimates, presented in table 7, reach a level of accuracy beyond what is possible using a calorimetric approach because they include intrinsically the inefficiencies and costs of biochemical conversion. The estimates derived in this paper, despite the crudeness of the approach and the omission of the biological costs, appear reasonable when compared to laboratory measurements. These estimates therefore provide useful working numbers for future research.

The estimates presented in table 7 can be used to assess the trade-offs made in tsetse physiology throughout the lifecycle between the generation of each of the principal metabolic products. For instance, it becomes possible to analyze the trade-off made by tsetse between the allocation of nutritional resources to the production of metabolic energy as against to the growth of the different components of biomass, muscle or fat. The estimates open up a new, quantitative strategy for reexamining the extensive analysis of digestive physiology in tsetse [20,21,53-56]. The estimates also provide a way to relate the extensive experimental evidence of respiration rates to the experiments of resource use and of changes in body mass. For example, the average respiration rate of mature male tsetse in the field has been measured based on the rate of loss of radioactive caesium isotopes [57,58]; those estimates can now be directly converted into estimates of blood mass use and metabolic energy consumption. The ultimate value of these estimates will become apparent once they are used to assemble a dynamic mass-energy budget which describes the time varying flows of biomass and energy in tsetse populations.

The biochemical approach to the estimation of conversion yields extends the work of ecologists interested in trophic interactions. The approach provides useful estimates which, despite the crude approach of the current study, can readily be refined using a similar analytic strategy but deriving the data from a systematic, experimental approach and using more sophisticated informatic tools. Nutritional ecologists working on digestive metabolism, like their counterparts working on cell metabolism, will be forced to use a biochemical approach in order to distinguish the purely biochemical costs of digestion from the biological costs. Ecologists developing organism specific dynamic mass-energy budgets will benefit from the detail provided by the biochemical approach. Population modelers working on physiologically based population models can use similar biochemical analyses to extend their mechanistic explanations to a more fundamental level and can chain models in multitrophic systems based on the actual currency, mass and energy, which drives ecological systems.

The results presented in this paper have been obtained solely for the purpose of developing an extensive analysis of tsetse and the disease which these flies transmit. The numbers provide the means to develop a dynamic mass-energy budget for tsetse through which to examine their feeding rate. The numbers also provide a quantitative basis from which to estimate the intrinsic rate of growth, in biomass terms, of tsetse populations. Jointly these provide a way to study of the rate of feeding of tsetse populations, which is the critical parameter determining the rate of transmission of trypanosomes and therefore the epidemiology of trypanosomiasis.

## Methods

The estimates presented in this paper were calculated by balancing the stoichiometry of all the biochemical reactions involved in the major metabolic pathways in tsetse based on published descriptions of the composition of the reactants, of the pathways used by tsetse metabolism, and of the specific reactions involved. These calculations required several simplifying procedures. Where multiple substrates could be used for the formation of a given end product, the substrate which needed the fewest biochemical steps to produce the desired end product is used. For instance, any amino acid can be used as a precursor to uric acid but here glycine (Gly), serine (Ser), cysteine (Cys) and alanine (Ala) are assigned to the conversion since they require the fewest biochemical steps. Where alternative intermediate pathways are possible, the most energetically efficient path which could be found was chosen. In all pathways, the costs of reactivation of the enzymes and catalysts are explicitly included in the resulting stoichiometric calculations.

A number of stoichiometric equivalences are used in this work. Guanosine triphosphate (GTP) is assumed energetically equivalent to adenosine triphosphate (ATP). The conversion of ATP to adenosine monophosphate (AMP) was assumed equivalent to two conversions of ATP to adenosine diphosphate (ADP). Similarly, reduced nicotinamide-adenine dinucleotide phosphate (NADPH) is assumed equivalent to reduced nicotinamide-adenine dinucleotide (NADH). Each of these is assumed to be energetically equivalent to 2.5 cystolic ATP whereas each reduced flavin-adenine dinucleotide (FADH_2_) is assumed equivalent to 1.5 cystolic ATP based on Michal [12](see page 44).

All calculations were performed using the free Gnumeric spreadsheet [59] and solved through iterative calculation. Since the proportions of conversion are independent of the actual quantity of substrate converted, each calculation was started, for convenience, with 100 moles of bloodmeal derived amino acids. The quantities of amino acids entering each metabolic pathway were assigned in a stepwise fashion, first to account for the excretion of the excess nitrogen through uric acid formation, next to form the ATP required, and finally to create the desired end products. Where the pathways required proportional formation of several end products, initial estimates for each path were changed iteratively to bring the end products to the correct proportions with the spreadsheet recalculating the metabolic consequences of these changes. The approach used here does not necessarily obtain the optimal solution since the biochemical pathways form a complex graph but this approach serves as a first approximation to the actual net proportion of conversion of substrates to products.

## List of abbreviations

**AA **Amino Acid.

**ADP **Adenosine diphosphate.

**AMP **Adenosine monophosphate.

**ATP **Adenosine triphosphate, the primary carrier of metabolic energy.

**CO**_2 _Carbon dioxide, also written CO2.

**-CoA **CoenzymeA, an enzyme that joins with metabolites in several biochemical reactions, for instance, acetyl-CoA is the complex of an acetyl radical and this enzyme.

**FADH**_2 _Flavin-adenine dinucleotide, a molecule used to transfer energy between the Krebs cycle and the ATP generating mitochondria.

**Fat **The fraction of body mass extracted by alcohol after desiccation.

**GTP **Guanosine triphosphate, an ATP equivalent carrier of matabolic energy.

**mol **Mole, Avogadro's number or 6.023 × 10^23^.

**NADH **Nicotinamide-adenine dinucleotide, a molecule used to transfer energy between the Krebs cycle and the ATP generating mitochondria.

**NADPH **Nicotinamide-adenine dinucleotide phospate, a molecule used to transfer energy between the Krebs cycle and the ATP generating mitochondria.

**O**_2 _Molecular Oxygen, also written O2.

**RDW **Residual Dry Weight, the remaining mass after desiccation and extraction of the alcohol soluble fraction. Here 'weight' is used as a synonym of mass, despite the slight inaccuracy.

**RNA **Ribonucleic acid.

### Amino acids

**Ala **Alanine

**Arg **Arginine

**Asp **Asparagine or Aspartic Acid

**Cys **Cysteine

**Glu **Glutamine or Glutamic Acid

**Gly **Glycine

**His **Histidine

**Leu **Leucine or Isoleucine

**Lyc **Lysine

**Met **Methionine

**Phe **Phenylalanine

**Pro **Proline

**Ser **Serine

**Thr **Threonine

**Try **Tryptophan

**Tyr **Tyrosine

**Val **Valine

## Authors' contributions

A. Custer performed all the research and analysis presented in the article and wrote the text.

## Supplementary Material

Additional File 1 **The Spreadsheet used for Estimation - Gnumeric Format**. This is the spreadsheet used to make the estimates in the text. This version of the file is in the file format used by the Gnumeric spreadsheet [59] which is simply a compressed (GNU zip) XML file. To view this file, download the Gnumeric software. Click here for download: Click here for file

Additional File 2 **The Spreadsheet used for Estimation - Excel Format**. This is the spreadsheet used to make the estimates in the text. This version of the file is in the Excel (TM) 97/2000/XP file format. Since this is a binary, unpublished format, Excel may complain on opening the file but the file should work. Click here for file

## References

[B1] Kooijman S (2000). Dynamic Energy and Mass Budgets in Biological Systems.

[B2] Nisbet R, Muller EB, Lika K, Kooijman S (2000). From molecules to ecosystems through dynamic energy budget models. Journal of Animal Ecology.

[B3] Kooijman S (2001). Quantitative aspects of metabolic organisation: a conceptual introduction. Philosophical Transactions of the Royal Society of London (B).

[B4] Gutierrez A, Baumgärtner J (1984). Multitrophic level models of predator-prey energetics: I. age specific energetics models -- pea aphid *Acyrthosiphon pisum *(harris) (homoptera: Aphididae) as an example. Candian Entomologist.

[B5] Gutierrez A (1996). Applied Population Ecology: A Supply-Demand Approach.

[B6] de Roos A, Persson L (2001). Physiologically structured models from versatile technique to ecological theory. Oikos.

[B7] Habtemariam T, Ruppanner R, Riemann H, Theis J (1983). An epidemiologic systems analysis model for African trypanosomiasis. Preventative Veterinary Medicine.

[B8] Gouteux J, Artzrouni M (1996). Faut-il ou non un contrôle des vecteurs dans la lutte contre la maladie du sommeil?. Bulletin de la Société de Pathologie Exotique.

[B9] Hershey D (1991). Digging deeper into helmont's famous willow tree experiment. American Biology Teacher.

[B10] Sterner R, Elser J (2002). Ecological Stoichiometry: The biology of elements and molecules to the biosphere.

[B11] Bursell E, Taylor P (1980). An energy budget for *Glossina *(Diptera: Glossinidae). Bulletin of Entomological Research.

[B12] Michal G (1999). Biochemical Pathways: An Atlas of Biochemistry and Molecular Biology.

[B13] Secor S, Diamond J (1997). Effects of meal size on postprandial responses in juvenile burmese pythons (python molurus). American Journal of Physiology: Regulatory, Integrative and Comparative Physiology.

[B14] Starck J, Moser P, Werner R, Linke P (2004). Pythons metabolize prey to fuel the response to feeding. Proccedings of the Royal Society of London (B).

[B15] Andersen J, Rourke B, Caiozzo V, Bennett A, Hicks J (2005). Physiology: Postprandial cardiac hypertrophy in pythons. Nature.

[B16] Fischer E, Sauer U (2003). Metabolic flux profiling of escherichia coli mutants in central carbon metabolism using gc-ms. European Journal of Biochemistry.

[B17] Fuhrer T, Fischer E, Sauer U (2005). Experimental identification and quantification of glucose metabolism in seven bacterial species. Journal of Bacteriology.

[B18] Edwards J, Palsson B (2000). Metabolic flux balance analysis and the in silico analysis of escherichia coli k-12 gene deletions. BMC Bioinformatics.

[B19] Gagneur J, Klamt S (2004). Computation of elementary modes: a unifying framework and the new binary approach. BMC Bioinformatics.

[B20] Hargrove J, Packer M (1993). Nutritional states of male tsetse flies (*Glossina *spp.) (Diptera: Glosssinidae) caught in odour-baited traps and artificial refuges: models for feeding and digestion. Bulletin of Entomological Research.

[B21] Loder P, Hargrove J, Randolph S (1998). A model for blood meal digestion and fat metabolism in male tsetse flies (*Glossinidae*). Physiological Entomology.

[B22] Bursell E (1965). Nitrogenous waste products of the tsetse fly, *Glossina morsitans*. Journal of Insect Physiology.

[B23] Lehane M (1991). Biology of Blood-sucking insects.

[B24] Bursell E, Billing K, Hargrove J, McCabe C, Slack E (1974). Metabolism of the bloodmeal in tsetse flies (a review). Acta Tropica.

[B25] Leak S (1999). Tsetse Biology and Ecology: Their Role in the Epidemiology and Control of Trypanosomiasis.

[B26] Buxton P, Lewis D (1934). Climate and tsetse flies: Laboratory studies upon *Glossina submorsitans *and *tachinoides*. Philosophical Transactions of the Royal Entomological Society London Series (B).

[B27] Bursell E (1958). The water balance of tsetse pupae. Philosophical Transactions of the Royal Society of London (B).

[B28] Ramos-Elorduy J, Moreno J, Prado E, Perez M, Otero J, de Guevara O (1997). Nutritional value of edible insects from the state of oaxaca, mexico. Journal of Food Composition and Analysis.

[B29] Cmelik S, Bursell E, Slack E (1969). Composition of the gut contents of third-instar tsetse larvae (*Glossina morsitans *Westwood). Comparative Biochemistry and Physiology.

[B30] Langley P, Pimley R (1979). Influence of diet on synthesis and utilization of lipids for reproduction by the tsetse fly *Glossina morsitans*. Journal of Insect Physiology.

[B31] Langley P, Pimley R (1979). Storage and mobilization of nutriment for uterine milk synthesis by *Glossina morsitans*. Journal of Insect Physiology.

[B32] Bursell E (1963). Aspects of the metabolism of amino acids in the tsetse fly, *Glossina *(Diperta). Journal of Insect Physiology.

[B33] McCabe C, Bursell E (1975). Interrelationships between amino acid and lipid metabolism in the tsetse fly, *Glossina morsitans*. Insect Biochemistry.

[B34] Bursell E, Slack E (1976). Oxidation of proline by sacrosomes of the tsetse fly, *Glossina morsitans*. Insect Biochemistry.

[B35] Bursell E (1977). Synthesis of proline by the fat body of tsetse fly (*Glossina morsitans*): metabolic pathways. Insect Biochemistry.

[B36] Bursell E (1978). Quantitative aspects of proline utilization during flight in tsetse flies. Physiological Entomology.

[B37] Bursell E, Downer R (1981). The role of proline in energy metabolism. Energy Metabolism in Insects.

[B38] Agosin M, Rockstein M (1978). Functional role of proteins. Biochemistry of Insects.

[B39] Cmelik S, Hurrell D, Lunat M (1969). Lipid content and composition of the tse-tse fly, *Glossina morsitans *Westwood. Comparative Biochemistry and Physiology.

[B40] Bursell E (1967). The excretion of nitrogen in insects. Advances in Insect Physiology.

[B41] McCabe C, Bursell E (1975). Metabolism of digestive products in the tsetse fly, *Glossina morsitans*. Insect Biochemistry.

[B42] Nation J (2002). Insect Physiology and Biochemistry.

[B43] Stevens L (1996). Avian Biochemistry and Molecular Biology.

[B44] Taylor P (1976). Blood-meal size of *Glossina morsitans *Westw. and *G. pallidipes *Austen (Diptera: Glossinidae) under field conditions. Transactions of the Rhodesian Scientific Association.

[B45] Bursell E (1961). Post-teneral development of the thoracic musculature in tsetse flies. Proccedings of the Royal Entomological Society of London (A).

[B46] Rogers D, Randolph S (1978). Metabolic strategies of male and female tsetse (Diptera: Glossinidae) in the field. Bulletin of Entomological Research.

[B47] Mathews CK, van Holde KE (1990). Biochemistry.

[B48] Rajagopal P, Bursell E (1966). The respiratory metabolism of resting tsetse flies. Journal of Insect Physiology.

[B49] Rajagopal P, Bursell E (1965). The effect of temperature on the oxygen consumption of tsetse pupae. Bulletin of Entomological Research.

[B50] Bursell E (1960). The effect of temperature on the consumption of fat during pupal development in glossina. Bulletin of Entomological Research.

[B51] Alberty R (1996). Calculation of biochemical net reactions and pathways by using matrix operations. Biophysical Journal.

[B52] Shastri A, Morgan J (2004). Calculation of theoretical yields in metabolic networks. Biochemistry and Molecular Biology Education.

[B53] Langley P, Wall R (1990). The implication of hunger in the tsetse fly, *Glossina pallidipes*, in relation to its availability to trapping techniques. Journal of Insect Physiology.

[B54] Randolph S, Rogers D, Kiilu J (1991). The feeding behaviour, activity and trappability of wild female *G. pallidipes *in relation to the pregnancy cycle. Medical and Veterinary Entomology.

[B55] Randolph S, Williams B, Rogers D, Connor H (1992). Modelling the effect of feeding-related mortality on the feeding strategy of tsetse (Diptera: Glossinidae). Medical and Veterinary Entomology.

[B56] Hargrove J (1999). Lifetime changes in the nutritional characteristics of female tsetse *Glossina pallidipes *caught in odour-baited traps. Medical and Veterinary Entomology.

[B57] Taylor P (1978). Radioisotopes as metabolic labels for *Glossina *(Diptera: Glossinidae). II. The excretion of ^137^Cs under field conditions as a means of estimating energy utilization, activity and temperature regulation. Bulletin of Entomological Research.

[B58] Hargrove J, Coates T (1990). Metabolic rates of tsetse flies in the field as measured by the excretion of injected caesium. Physiological Entomology.

[B59] GNOME (2004). The Gnumeric Spreadsheet. http://www.gnome.org/projects/gnumeric/.

